# Role of traditional and new biomarkers in breast carcinogenesis

**DOI:** 10.3332/ecancer.2009.157

**Published:** 2009-10-29

**Authors:** D Macis, M Cazzaniga, A De Censi, B Bonanni

**Affiliations:** 1 Division of Cancer Prevention and Genetics, European Institute of Oncology, via Ripamonti 435, 20141 Milan, Italy; 2 Division of Medical Oncology, E O Ospedali Galliera, Genoa, Italy

## Abstract

In recent decades, several biomarkers have been investigated as predictors of breast cancer risk, development, prognosis and treatment efficacy.

The detection of biomarkers strongly associated with breast carcinogenesis has an enormous potential, especially for selecting subjects at high risk of developing breast cancer who could benefit from chemopreventive treatments.

Although the number of potential biomarkers continues to increase, a unique biomarker for breast cancer risk prediction has not been identified and it is probable that a panel of biomarkers will prove optimal. Further studies are needed to validate breast cancer biomarkers evaluation for individual risk assessment.

This review summarizes the main biomarkers, which are important at different stages of breast carcinogenesis with updates from the recent literature.

## Introduction

Efficacy in breast cancer early diagnosis, treatment and prevention is a big challenge considering that breast cancer is the most common diagnosed cancer in women worldwide. Biomarkers have a critical role both in monitoring cancer progression and assisting the identification of high-risk subjects. The general characteristics of a biomarker should include high reproducibility and detectability, easy collection from patients by minimally invasive techniques and high correlation with the disease with different expression in healthy versus affected subjects.

An ideal biomarker for monitoring cancer treatment should be strongly correlated with cancer growth and should be an index for drug efficacy. A good candidate biomarker for cancer prevention would be a marker directly correlated with risk and with molecular mechanisms of carcinogenesis in pre-cancerous tissues, should be differentially expressed in average versus high-risk populations and should predict response to chemopreventive agents. Several tumour markers have shown evidence of clinical usefulness and have been recommended for use in practice in breast cancer patients: CA 15-3, CA 27.29, carcinoembryonic antigen (CEA), oestrogen and progesterone receptors (ER, PR), human epidermal growth factor receptor 2 (HER-2), urokinase plasminogen activator (uPA), plasminogen activator inhibitor 1 (PAI-1) and certain multiparametre gene expression assays (Oncotype DX, MammaPrint, Rotterdam Signature) [1].

In the last decade, much effort has been focused on discovering biomarkers that can be used to predict breast cancer risk. Among the established risk biomarkers, deleterious germline mutations in BRCA1/BRCA2/TP53 genes have been demonstrated as strong predictors of breast cancer development [2] and recent studies have also investigated the possible association between single nucleotide polymorphisms (SNPs) and breast cancer risk [3–5]. An association between levels of endogenous sex hormones with breast cancer risk have been shown in post-menopausal women [6,7], whereas other hormones and circulating biomarkers such as serum IGF-1, IGFBP-3 and also testosterone have been demonstrated to be associated with risk, especially in pre-menopausal women, although more recent data have also demonstrated relevance in post-menopause [8–12].

Other potential biomarkers that have been investigated for an association with breast cancer include hormonal and nuclear receptors, membrane receptors and signal transduction factors, anti-inflammatory and antioxidant factors, apoptosis and angiogenesis factors, proliferation markers and antigens, epigenetic modulation factors, cellular inducible enzymes, cell cycle regulators, oncogenes and tumour suppressor genes [13,14].

In this review, we will discuss the main biomarkers which have been reported to have a role in breast cancer early development and progression.

### Hormonal and nuclear receptors

Oestrogen and progesterone are essential hormones for breast growth, and their receptors (ER and PR) are established predictive factors for breast cancer treatment efficacy and disease outcome. ERs are transcription factors, which mediate the action of oestrogens, whereas the PR gene is regulated by oestrogen, and so the PR could be a marker of oestrogen action in breast cancer [15].

Previous studies have shown that ER and PR levels are associated with a favourable breast cancer prognosis and are highly predictive of benefit from endocrine treatment in both the adjuvant and metastatic settings [16–18]. Two different isoforms for each receptor have been described: ERa· and ERb; PRA and PRB. Functional experiments have demonstrated that ERa and ERb have completely different roles in breast cancer: ERa acts as a tumour promoter, whereas ERb is a tumour suppressor. The presence of ERb in breast tumours is associated with better prognosis and longer disease-free survival [19]. ERa expression has been demonstrated to be increased in normal epithelium taken from a tumour-bearing breast [20], and another study reported increased ERa expression in the tissue of women at high risk of developing breast cancer [21]. However, it is important to consider the relative amounts of ERa and ERb; as normal breast tissue becomes tumourigenic, the amount of ERa increases whereas the amount of ERb decreases [22].

In normal breast, PRA and PRB are co-expressed at similar levels on luminal epithelial cells, suggesting that both proteins are required to mediate physiologically relevant progesterone signalling, and an imbalance in the native ratio of the two isoforms may lead to alterations in PR signalling [23,24]. Previous studies have demonstrated that the ratio between PRA and PRB is altered during the breast carcinogenesis process [25,26].

Androgen receptors (AR) are expressed by the majority of human breast carcinomas, at a frequency comparable to or higher than that reported for ER and PR [27,28]. AR shows a statistically significant association with important clinical and pathologic prognostic factors [29], such as histopathological grade, tumour invasiveness and axillary lymph node involvement [30–33]. Furthermore, some studies reported statistically significant associations between increased levels of testosterone and higher breast cancer risk in both pre- and post-menopausal healthy women [11,34–36], supporting a role for androgens and their receptors in breast carcinogenesis.

All these findings highlight the role of ER, PR and AR in promoting breast cancer and consequently their potential as drug targets in both preventive and therapeutic settings.

Other members of the steroid/thyroid hormone receptor super family include the nuclear retinoid receptors. Receptor selective retinoids inhibit the growth of both normal and cancerous human breast cells predominantly through induction of a G1 cell cycle block [37]. There are two classes of retinoid receptors: retinoic acid receptors (RARs) and retinoid X receptors (RXRs). Both types of receptors are encoded by three distinct genes a, b and g. Their functional activities require dimerisation with a member of the nuclear non-steroidal receptor family, in particular RXRs dimerise with peroxisome proliferator-activated receptors (PPARs) and with the vitamin D receptor (VDR) [38–40].

The RARs and RXRs are thought to mediate the effects of retinoids on cell growth, differentiation and apoptosis and, therefore, to have a role in mammary carcinogenesis suppression [41–43]. RARa expression has been demonstrated to be positively linked to proliferative activity and to ER expression [44], whereas RARb may act as a tumour repressor gene, since its expression is progressively lost with increasing proliferative activity of the tumour [45]. Furthermore, an increasing RARa/RARb ratio may be a marker of progression from the pre-invasive to the invasive state in breast cancer [46].

The PPARs are also members of the nuclear receptor super family, which includes steroid, retinoid and thyroid hormone receptors [47,48]. Three isoforms of PPAR have been identified, a, d and g. PPARg has been found to be expressed in normal breast epithelium and breast cancers [49]. It heterodimerises with RXR and then binds to the peroxisome proliferator response elements in the promoter regions of targets genes, allowing for activation of gene transcription that is responsible for cell cycle modulation, cellular differentiation, decreased proliferation and inhibition of angiogenesis [50,51].

Previous studies suggested that PPARg signalling may exacerbate mammary gland tumour development in mice [52] and PPARg ligands have been shown to inhibit the proliferation and induce the apoptosis of breast cancer cell lines *in vitro* through the activation of PPARg [53]. Furthermore, a favourable impact of PPARg expression on disease-free survival of patients with ductal breast carcinoma, and its possible cooperation with ERb in exerting that favourable effect has been reported [54].

All these results document important roles for retinoid receptors and PPARs in oncogenesis, thereby identifying new targets for cancer therapy and prevention. Further evidence supporting the importance of the retinoid pathway in breast cancer development has been provided by a large randomised trial with the synthetic retinoid fenretinide that has shown a reduced risk of recurrence after primary surgery in pre-menopausal women [55].

The vitamin D receptor is a nuclear receptor present on over 80% of human breast cancers and mediates the action of 1,25-dihydroxy-vitamin D_3_, the biologically active form of vitamin D, that modulates differentiation, cell cycle and apoptosis of stromal and epithelial cells derived from mammary gland and breast cancers *in vitro* [56–60]. A vitamin D analogue significantly enhanced the ability of tamoxifen to prevent mammary tumours in rats, suggesting that vitamin D compounds and anti-oestrogenic compounds might protect against breast cancer through independent mechanisms [61].

The expression patterns of the heterodimers formed by VDR and RARs or RXRs have been studied by immunohistochemistry in benign and malignant human breast tissues. Higher levels of all receptor types were found in both *in situ* and infiltrative carcinomas compared to benign breast diseases or normal breast tissue [62,63]. Previous studies on VDR expression and its relationship with breast cancer prognosis indicated a longer disease-free survival in patients with VDR positive tumours compared to those with VDR negative neoplasms [60,64]. An inverse correlation between vitamin D intake and breast cancer risk has been observed in several studies [65–67] and also low serum 1,25-dihydroxy-vitamin D_3_ has been correlated with increased breast cancer risk and metastasis [68,69].

Several epidemiologic, mechanistic and experimental data support the chemopreventive potential of vitamin D and its analogues, although hypercalcemic side effects represent a major obstacle to their clinical development. A proposed strategy for using a non-calcemic dose in the chemopreventive setting is to administer vitamin D or its analogues in combinations with other agents, such as retinoids or anti-oestrogenic compounds [70].

### Membrane receptors and signal transduction factors

Tyrosine kinase signalling has been demonstrated to have an important role in the genesis and progression of human breast cancer as well as in all the steps of the normal mammary gland development [71]. Receptor tyrosine kinases (RTK) belong to a class of transmembrane receptors composed of nearly 60 members distributed among 20 subfamilies. A little more than half of the known RTKs have been repeatedly found in either mutated or over-expressed forms to be associated with human malignancies, including sporadic cases [72,73]. The most studied receptors of this super family are the human epidermal growth factor receptors, which belong to subclass I, comprising four members (HER1, HER2, HER3, HER4). The epidermal growth factor receptor (EGFR or HER1) has been demonstrated to be involved in malignant transformation and cancer progression and its over-expression in cancer tissues has been associated with a more aggressive phenotype and a worse survival [74,75]. Nearly 45% of human breast tumours express EGFR and previous studies reported an inverse correlation between EGFR and ER expression in breast cancer [76,77]. EGFR and ER are also frequently co-expressed in normal-appearing cells obtained from breast cancer patients [78]. Moreover, Fabian *et al* found a significantly higher expression of EGFR in ductal cells of needle aspirates from women at high risk for breast cancer development than in low-risk control subjects [79], suggesting EGFR as a viable target for breast cancer chemoprevention.

The HER2 gene is amplified and over-expressed in about 25% of breast cancers, conferring a more aggressive biology [80]. Over-expression of the HER2 gene is associated with rapid tumour growth, increased risk of recurrence after surgery, poor response to conventional chemotherapy and shortened survival [81].

It has been reported that tamoxifen treatment up-regulates HER2 by 40% in breast cancer cells expressing both HER2 and ER [82], and that when HER2 is co-expressed with ERa, the patients show a shorter disease-free survival and overall survival [83]. These findings suggest that HER2 modulation is an ER-mediated process.

The other two members of the HER family, HER3 and HER4, are of particular interest because of their ability to interact directly with HER2. A recent study presented striking evidence that alterations in HER3 exist in breast cancer [84] and another study demonstrated that blocking HER2 resulted in anti-proliferative effects accompanied by a decrease in HER3 signalling activity [85]. Furthermore, HER3 contributes to HER2-associated tamoxifen resistance and a decrease in HER3 levels restores sensitivity to tamoxifen [86] and gefitinib (an EGFR tyrosine kinase inhibitor) has been demonstrated to decrease EGFR and HER3 phosphorylation through the inhibition of EGFR/HER3 dimerisation [87]. HER4 protein over-expression has previously been described as a positive prognostic factor in breast tumours [88–90]. HER4 amplification detected by FISH was found to be correlated with a positive oestrogen receptor status [84,91], whereas conflicting conclusions were reported by other authors who found that HER4 amplification and ER activity were negatively correlated [92]. These differences may be due to the variable responses by HER4 to its activating ligand Heregulin, resulting in either proliferation or differentiation, and perhaps influenced by homodimerisation or heterodimerisation with other HER family members [93].

Two other membrane receptors demonstrated to have a role in breast carcinogenesis are the transforming growth factor beta (TGF-b) receptor and the insulin-like growth factor (IGF) receptors. TGF-b signalling pathway is implicated in cell senescence, regulation of cell growth and differentiation [94]. TGF-b has a dual role: in normal development, it inhibits cell proliferation by induction of apoptosis and cell cycle arrest and promotes cell differentiation, whereas during tumourigenesis TGF-b secreted from tumour cells often loses its inhibitory function in favour of its oncogenic activity [95]. Increased production of TGF-b occurs in different tumour types and correlates with the severity of the tumour grade [96,97]. Since the role of TGF-b pathway in carcinogenesis has been elucidated, specific molecular inhibitors, such as TGF-b antibodies and antisense oligonucleotides that block TGF-b receptor have been investigated in pre-clinical and clinical studies for cancer therapy [98,99]. Recent studies demonstrated the efficacy of TGF-b inhibitors in reducing the *in vivo* bone metastatic capacity of human breast cancer cells [100–102].

Another recent study reported that TGF-b is a potential mediator of retinoic acid and tamoxifen-induced apoptosis in breast cancer cell lines, suggesting that the TGF-b pathway may have a role also in the breast chemoprevention setting [103].

The IGFs are peptide hormones involved in regulation of cell proliferation, differentiation and apoptosis [104]. Several studies investigating the relationship between the circulating concentration of IGF-I and IGFBP-3 and breast cancer risk demonstrated a correlation between increasing IGF-I and IGFBP-3 levels and increased breast cancer risk, especially in pre-menopausal [8–10] and more recently also in post-menopausal women [12]. Anti-oestrogens, effective in breast cancer treatment and prevention, reduce serum IGF-I levels [105]. IGF-I receptors are over-expressed in many breast cancers [106], and recent clinical trials have investigated the effectiveness of IGF-I receptor inhibitors in cancer therapy [107]. Circulating IGF-I mainly binds to IGFBP-3, a protein that regulates the mitogenic actions of IGFs and inhibits their apoptotic effect and also has an IGF-independent inhibitory effect on cell growth [104]. Baxter and co-worker showed that IGFBP-3 production is potently induced by TGF-b and proposed a role for IGFBP-3 in mediating TGF-b inhibitory activity [108,109]. IGFBP3 is also a ligand for the nuclear RXR-a receptor, which was shown to be necessary for IGFBP-3 induced apoptosis [110]. Results from a phase III breast cancer prevention trial with fenretinide, suggested that the reduction of IGF-I and IGFBP-3 levels may in part explain the cancer risk reduction observed in women ≤50 years of age [111].

### Inflammation and oxidation markers

Cyclooxygenase-2 (COX-2) is an important inflammatory mediator, responsible for the conversion of arachidonic acid into prostaglandins. In the last decade, several studies reported a correlation between COX-2 expression and breast cancer. Elevated COX-2 protein levels have been detected in approximately 40% of invasive breast cancers and COX-2 over-expression has been demonstrated to correlate with large tumour size, high grade, high proliferation, hormone receptor negative status and over-expression of HER-2 [112–117]. The role of COX-2 in breast cancer pathogenesis suggested the COX-2 signalling as a target for breast cancer treatment and prevention. Some meta-analyses and case control studies reported a moderate reduction in risk of breast cancer, up to 24%, with non-steroidal anti-inflammatory drugs (NSAIDs) [118–120]. Pre-clinical studies suggested that celecoxib, which is a selective inhibitor of COX-2, is effective in both preventing and treating breast cancer in a dose-dependent manner [121,122]. However, other trials have identified an increased cardiovascular risk associated with COX inhibitors, probably due to their selective depression of prostacyclin levels [123]. Sauter *et al* investigated the possible correlation between celecoxib administration and prostaglandin concentration in serum and nipple aspirate fluid (NAF), reporting a reduction of prostaglandin levels in NAF of post-menopausal high-risk women and a reduction in both NAF and plasma prostaglandin levels in women with newly diagnosed breast cancer [124]. Further clinical trials are necessary to better understand the chemopreventive and treatment potential of celecoxib.

Matrix metalloproteinases (MMPs) are a family of proteases involved in the regulation of the cell microenvironment, matrix turnover, growth factor bioavailability and several aspects of immunity and inflammation. Other classes of proteases, which interact with MMPs are a disintegrin and metalloproteinases (ADAMs), and tissue inhibitor of metalloproteinases (TIMPs) [125,126]. Epidemiological evidence suggests an association between inflammation and breast cancer and several studies investigating the role for MMPs, ADAMs and TIMPs in breast cancer risk and progression have demonstrated that high levels of MMP9 are associated with poor breast cancer prognosis [127,128]. MMP1 may prove be a breast cancer risk marker since it has been found in tissues with atypical ductal hyperplasia and in ductal lavage cells from patients at risk for developing breast cancer [129]. Other studies reported that TIMP1 levels correlate positively with higher serum HER-2 levels, increased metastasis and reduced survival in breast cancer patients [130], whereas TIMP3 over-expression was associated with successful adjuvant endocrine therapy, good prognosis and longer disease-free survival [131–133]. Some evidence also suggests a role for TIMP3 as a breast cancer risk marker as TIMP3 levels were higher in mammographically dense breasts, which are considered to be at higher risk for developing breast cancer [134]. Although the biology of ADAMs is less understood than that of MMPs, several components of this family have been found in breast cancer and, in particular, ADAM9 correlates positively with HER-2 levels [135] and with positive response to tamoxifen [136]. Furthermore, ADAM12 urine levels have been found to positively correlate with breast cancer progression, suggesting a possible diagnostic role [136].

C-reactive protein (CRP) is an acute-phase protein, synthesized in the liver, which is considered a marker of inflammation [137]. CRP serum levels have been associated with breast cancer and increased circulating levels of CRP have also been detected in more advanced stages of breast malignancy [138], suggesting the potential use of CRP as a biomarker of disease prognosis [139]. Elevated levels of CRP are associated with reduced survival in breast cancer patients [140–142]. In the Rotterdam Study, a statistically significant increase in breast cancer risk associated with higher CRP levels at baseline was reported [143], whereas other authors did not find any significant association between circulating CRP and breast cancer risk [144–146]. In a recent study, the relation between the level of CRP protein in NAF and breast cancer risk as predicted by the Gail model was investigated. CRP was found in NAF samples, and it was not significantly related to serum CRP levels, suggesting that CRP presence in NAF may reflect the early development of proliferative changes in the ductal epithelium preceding carcinoma *in situ* and invasive carcinoma [147].

Osteopontin (OPN) is a phosphoglycoprotein involved in a variety of physiopathologic processes such as bone remodelling, angiogenesis, inflammatory response, cell growth and tissue differentiation and wound healing [148]. OPN positivity of the primary breast tumour is significantly associated with decreased survival [149,150] and recent studies by Mirza *et al* suggested that the OPN-c splice variant could be a selective and prognostic marker for human breast cancer, as OPN-c mRNA was identified in 80% of breast carcinomas [151]. Patani *et al* demonstrated a significant positive association of OPN-b and OPN-c expression with adverse pathological and clinical outcomes [152].

In a cohort of women with newly diagnosed metastatic breast cancer, high-baseline OPN plasma levels or OPN increase > 250 ng/ml at any time during follow-up were found to be associated with poor survival [153] and higher OPN levels in patients with metastatic breast cancer may be associated with an increased number of involved sites and decreased survival [154].

### Apoptosis, cell cycle regulators and angiogenesis factors

The apoptosis-signalling pathway has an important role in cancer onset and progression. Many apoptotic proteins have been studied for an association with breast cancer pathogenesis. The inhibitors of apoptotic proteins are a more recently described family of proteins that function as endogenous inhibitors of caspases [155]. Survivin is the only member of this family, which has a dual role in inhibition of apoptosis and regulation of mitosis [156]. Data from a large analysis of human transcripts reported a higher expression of survivin in cancer tissues compared with normal tissues [157]. In an immunohistochemical study, survivin over-expression was detected in surgically resected primary tumour specimens of breast cancers [158], and recent studies reported that survivin is linked to aggressive breast cancers [159], resistance to apoptosis [160] and modulation of HER-2 signalling [161]. Few studies investigated the possible association between survivin and histological parameters or prognostic factors with controversial results: some of them reported no association between tissue and serum survivin expression and tumour size, p53 expression, oestrogen and progesteron receptors levels [162–164], while others showed that high-tissue survivin expression was correlated with high-nuclear grade, negative hormone receptor status and HER-2 over-expression [165], and that the presence of survivin is a highly significant independent predictor of shorter duration of survival in patients with poor prognostic features [166].

TP53 is a tumour suppressor gene that encodes the p53 protein, which is activated in response to several forms of cellular stress and mediates many cellular processes, including cell cycle arrest, apoptosis, senescence and differentiation [167,168]. Several studies have investigated the predictive value of TP53 mutations for tumour response to treatment and patient outcome in various cancers and for breast cancer the presence of a mutation was reported to correlate with shorter survival or a poor outcome [169,170]. TP53 mutation status was also shown to be useful to identify women at higher risk of disease recurrence and death when their tumour had a HER2 gene amplification [171].

P53 has been shown to be a possible target of the immune system. Antibodies against p53 have been detected in different types of malignant disease, with the highest positivity rates observed for breast cancer [172]. There are studies reporting a significant correlation between presence of p53 antibodies and the detection of mutated p53 protein in tissue sections and higher serum levels of antibodies in breast cancer cases than in healthy subjects from the general population. Furthermore, in breast cancer patients the presence of p53 antibodies also indicated a shortened survival [173–175].

Cyclin D1 is a key factor involved in the regulation of the G1-S phase transition during the cell cycle. Cyclin D1 over-expression has been observed in DCIS, suggesting a possible role in breast cancer development [176–178]. Previous studies have reported that in ER-positive breast cancer patients, cyclin D1 over-expression is associated with overall and relapse-free survival [179,180].

It has been shown that cyclin D1 can activate oestrogen receptor transcriptional activity in the absence of estradiol, and that this activity can be inhibited by 4-hydroxytamoxifen [181]. Furthermore, published *in vitro* data suggested that retinoids reduce cyclin D1 expression in human breast cancer cell lines [182,183]. These data are of great interest, since fenretinide has shown to induce a significant risk reduction of second breast cancer in pre-menopausal women, suggesting a possible role as breast cancer chemopreventive agent in high-risk young women [184].

Vascular endothelial growth factor (VEGF) is a key enhancer factor of angiogenesis, the process of new blood vessels formation, involved in cancer development and progression. Several studies have investigated the association between VEGF expression in cancer tissues and plasma with breast cancer. High levels of VEGF expression in breast cancer tissues have been associated with poor prognosis and decreased overall survival [185,186], and many authors have reported that circulating VEGF levels are increased among breast cancer women [187–189]. It has been suggested that VEGF expression may help predict the biologic aggressiveness of DCIS [190]. VEGF over-expression was found not only in DCIS, but also before remodelling of the fibroblastic stroma of pre-malignant breast lesions [191]. Furthermore, previous studies evaluating VEGF expression and angiogenesis in breast cancer patients, have reported that sex steroids regulate angiogenesis and increase VEGF expression [192], whereas the agonist of the oestrogen receptor tamoxifen has been shown to decrease VEGF expression and angiogenesis [187,189,193].

### Proliferation markers and antigens

Several studies have been conducted to investigate the possible use of proliferation markers as breast cancer prognostic indicators. Various methods are available for the measurement of proliferation rates in tumours, including mitotic counts, estimation of the fraction of cells in S-phase of the cell cycle and proliferation-associated antigens [194]. A previous study reported that mitotic count was a stronger predictor of survival than tumour size and lymphatic or skin invasion [195]. However, one limitation of mitotic index as a measure of proliferation is that the duration of the mitotic phase of the cell cycle is variable and consequently the correlation of number of mitoses and proliferation rate is not necessarily linear [196]. The measurement of the fraction of cells in chromosomal DNA synthesis (the S-phase of cell cycle) is one of the standard methods of assessing proliferation, with various techniques that can detect DNA replication. Recently, alternative methods of assessing proliferation that are based on the detection of nuclear antigens by immunohistochemistry have been investigated. Ki67, a protein expressed in the nucleus during the cell cycle, is actually used as measure of proliferation [194]. It has been demonstrated that Ki67 correlates significantly with estimates of the mitotic count index and the measurement of the fraction of cells in S-phase [197,198]. Several studies reported an association between Ki67 and disease-free and overall survival, with an increased risk of recurrence in tumours with a high Ki67 [199–201]. Several authors measured Ki67 in tamoxifen-treated breast cancer patients, all reporting a decrease in Ki67 [202–204]. Ki67 has been validated as a surrogate endpoint biomarker reflecting the superiority of anastrozole versus tamoxifen in early stage breast cancer, with a greater reduction in Ki67 being achieved with anastrozole [205]. In recent studies, Ki67 was measured either in random periareolar fine needle aspiration and in ductal lavage samples from women at high risk of developing breast cancer, and it was reported a significant association between Ki67 expression and atypia and cell yield, indicating Ki67 as a surrogate biomarker in early-phase chemoprevention trials [206,207].

Proliferating cell nuclear antigen (PCNA), a nuclear protein involved in DNA repair processes, has been investigated, but appear to correlate poorly with Ki67 and mitotic count, suggesting a more limited use in assessing proliferation [208].

## Conclusions

The biomarkers that have been discussed in this review are involved at different stages of the breast carcinogenesis multi-step process. Each of the described biomarkers may be a target for drug treatment or prevention. Notwithstanding, we should take into account that the absolute benefit of a treatment varies greatly between individuals. Biomarkers may also be helpful for identification of high-risk subjects who will benefit from a given treatment. However, the currently available data do not support the implementation of a cancer risk model based on biomarkers evaluation. Further studies and prospective clinical randomised trials are necessary to better define whether and which biomarkers could be used in clinical practice for better treatment selection and patient follow-up.
